# Personalization of the Learning Path within an Augmented Reality Spatial Ability Training Application Based on Fuzzy Weights

**DOI:** 10.3390/s22187059

**Published:** 2022-09-18

**Authors:** Christos Papakostas, Christos Troussas, Akrivi Krouska, Cleo Sgouropoulou

**Affiliations:** Department of Informatics and Computer Engineering, University of West Attica, 12243 Athens, Greece

**Keywords:** adaptive learning, spatial ability, augmented reality, personalized system, fuzzy weights

## Abstract

Adaptive systems and Augmented Reality are among the most promising technologies in teaching and learning processes, as they can be an effective tool for training engineering students’ spatial skills. Prior work has investigated the integration of AR technology in engineering education, and more specifically, in spatial ability training. However, the modeling of user knowledge in order to personalize the training has been neither sufficiently explored nor exploited in this task. There is a lot of space for research in this area. In this work, we introduce a novel personalization of the learning path within an AR spatial ability training application. The aim of the research is the integration of Augmented Reality, specifically in engineering evaluation and fuzzy logic technology. During one academic semester, three engineering undergraduate courses related to the domain of spatial skills were supported by a developed adaptive training system named PARSAT. Using the technology of fuzzy weights in a rule-based decision-making module and the learning theory of the Structure of the Observed Learning Outcomes for the design of the learning material, PARSAT offers adaptive learning activities for the students’ cognitive skills. Students’ data were gathered at the end of the academic semester, and a thorough analysis was delivered. The findings demonstrated that the proposed training method outperformed the traditional method that lacked adaptability, in terms of domain expertise and learning theories, considerably enhancing student learning outcomes.

## 1. Introduction

Extended Reality (XR) is an all-encompassing term that combines the experiences of augmented reality (AR), virtual reality (VR), and mixed reality (MR), meaning all the technologically enhanced realities fall under the umbrella term of XR. AR uses the existing real-world environment and puts virtual information on top of it to enhance the experience. On the contrary, VR immerses users into an entirely different environment, typically a virtual one created and rendered by computers. Finally, MR is a user environment in which physical reality and digital content are combined in a way that enables interaction with and among real-world and virtual objects.

AR technology superimposes a computer-generated 3D image on a user’s view of the real world [1]. AR is an enhanced version of the real physical world that is achieved through the use of digital stimuli, such as visual and/or sound elements, and delivered via technology. AR applications are user-friendly, providing a straightforward and pleasant method of human–computer interaction. AR has been integrated in many fields, gaining much potential in educational settings, and more specifically, in engineering training [2]. 

The improvement of students’ visualization skills is crucial in engineering education for the growth of their design abilities [3]. Numerous studies have examined the benefits of AR technology for enhancing students’ engineering drawing efficiency, as well as for improving their spatial skills, which are crucial for their studies and future careers [4,5,6,7]. Although the integration of AR in spatial ability training has been explored by many researchers, there is no research specifically designed as a personalized AR spatial ability training system that considers the student profile [8].

Personalized training offers great pedagogical affordances, as it provides an enhanced learning experience, improves student engagement, and promotes knowledge acquisition [9]. The adaptive systems integrate built-in components in order to offer knowledge domain adaptivity and deliver different learning activities, tailored to the student’s profile. The learning material’s pedagogical potential increases with how adaptable it is to the cognitive needs and capabilities of the students. For instance, in the case of a student who studies a specific domain concept, having a high knowledge level, and has been given many learning activities that are not appropriate for that level, then the learning process may not go as expected and the student would feel frustrated [10]. 

During the last years there has been a trend in combining mobile technologies with AR to achieve the implementation of AR applications that benefit from the portability features and immediate access to information that are achieved with mobile devices [11]. However, the combination of AR and its application in educational settings remains an open area research. There are not specific guidelines for the description of educational content based on AR techniques or methodologies for the design and creation of highly interactive material so that it can provide personalized learning in any place and at any time. The integration of such applications in an adaptive and accessible learning process would allow students to present highly interactive content, personalized to their characteristics and needs, and as such, can interpret the contents and relate them to the real world.

The main contributions of the paper can be identified as follows:

- The use of fuzzy logic in an AR system in engineering education.
- The use of the Structure of the Observed Learning Outcomes (SOLO) for the instructional design of learning content.
- The delivery of the learning activities taught to students in an AR system.

The novelty of our research is the integration of adaptive techniques and learning theory to provide students with personalized learning activities within the framework of spatial ability training through AR. Fuzzy weights are specifically used in a rule-based decision-making engine to provide engineering students with adaptive learning activities, in terms of their knowledge level. The research question that the manuscript seeks to answer is how effective fuzzy weights can model the learner’s knowledge level in an AR training system.

Our paper is structured as follows. In Section 2, the literature review is investigated. Section 3 presents the system’s instructional design. Section 4 describes the matching between the learning activities and the normalized fuzzy weights. Section 5 presents the system’s evaluation, and finally, Section 6 concludes the work and continues with the limitations and future work.

## 2. Literature Review

The literature review reveals numerous studies focusing on the integration of AR technology in fields such as education [12,13,14], tourism [15,16,17], industry [18,19], marketing [20,21,22], and medical [23,24]. 

Many studies have explored the positive effects of AR technology in education, as compared to the traditional methods of teaching and learning. Previous authors [13] tested a firefighting training system using AR, which was more cost-effective and safer, compared to large-scale real-life training. Simulating firefighting scenarios helped the trainees evaluate their knowledge and deal with risky circumstances.

Another study [16] presented a mobile AR travel guide, supporting personalized recommendations. The authors explored the relationship between system properties, user emotions, and adoption behavior. More specifically, the developed AR application built a user profile, based on users’ preferences, and according to the updated profile, users are offered extra media features.

AR is considered one of the most disruptive technologies in the field of marketing [21]. Consumers derive tangible benefits from AR technology and expect it as part of their purchasing process. AR has been incorporated into store catalog applications, which allow consumers to visualize what different products would look like in different environments. With AR technology, marketers are able to carry out successful digital campaigns. AR aids marketing as it can let the customers try before they buy, augment touring and assistance, and finally, augment branding materials. 

In [23], the authors explored the potential of VR, AR, and MR in both dental education and clinical dentistry. AR and VR technology can be beneficial not only for dental students, but also for patients, as AR and VR can reduce dental anxiety and treat dental phobias. Furthermore, a systematic review [24] investigated the usability of AR in the area of health sciences on the aspect of the psychopedagogy of students in higher education. The usage of AR improved many aspects of the learning process, including motivation, satisfaction, and autonomous learning [25,26]. 

Based on the analysis of the relevant literature, there are different aspects regarding the adaptation technique, which are mainly based on (a) the student’s specific learning style [27,28], (b) the student’s thinking preference [29,30,31], (c) information processing capability of the student [32], (d) student motivation [33,34], (e) the learning path [35,36,37], and (f) the learning material [38,39]. However, their adaptation focuses primarily on the manner in which the domain knowledge is presented, rather than the quantity and type of learning activities that are offered to the students. These studies suggest that students’ knowledge levels are the most important determinant. The field of adaptive domain knowledge models is poorly explored, and research has not yet focused on maintaining learner engagement [40,41].

In view of the above, it is concluded that previous research has not focused on the adaptivity of domain knowledge in terms of the scope and nature of the learning activities taught to students. Furthermore, past studies related to domain knowledge adaptivity have not utilized fuzzy weights in decision making to achieve adaptivity and did not incorporate learning theories to maintain pedagogical affordability. There is scope for intelligent tutoring techniques in engineering education.

The rest of this work initially describes the instructional design, which is a novelty in AR spatial ability training systems. Then, we analyze the fuzzy weights membership functions of the current knowledge level, and how the output determines how many learning activities will be delivered to the learner, transforming the system into an adaptive one. This structure makes it easier for the reader to navigate the paper and understand the material presented. 

## 3. Instructional Design

The domain knowledge included learning activities where students manipulate (move, rotate, zoom) augmented 3D objects to better understand the object geometry, and finally, design the technical drawing of their orthographic views. The domain knowledge consists of fifteen chapters related to the technical drawing course, providing an introduction to advanced sections on complexity.

A guiding framework suitable for the development of educational products, namely the Structure of the Observed Learning Outcomes (SOLO) model [42,43], was used for the instructional design of the learning activities [44,45].

The SOLO taxonomy describes the levels of students’ understanding in ascending order of complexity. In particular, the model consists of five levels of understanding, namely pre-structural (L1), unistructural (L2), multi-structural (L3), relational (L4), and extended abstract (L5). This model can be used to deliver the most appropriate learning activity to students with the intention of improving their learning outcomes [46]. Therefore, our Personalized Augmented Reality Spatial Ability Training application (PARSAT) uses the SOLO model to offer each student the learning activities that suit them best. Table 1 illustrates the learning goals and the corresponding activities per SOLO level.

## 4. Adaptation of the Learning Activities

### 4.1. Fuzzy Weights

The users of PARSAT are learners with a variety of background knowledge in the domain of technical drawing, and therefore, they have different learning requirements. In order to provide each learner with the most accurate learning path and material, an AR system was developed (namely PARSAT), which determines their knowledge level and their learning needs. This is accomplished through the student model, which, nowadays, is present in the majority of most adaptive educational software [49]. 

The student model’s function is to represent the students’ current level of knowledge, and it is crucial for the system to provide the appropriate personalization for students’ learning needs [50]. There are other methods that can be used to construct the student model, including neural networks and fuzzy logic networks [51]. The fuzzy weightings that define students’ present level of knowledge, are the foundation of the PARSAT student model. Each student’s degree of knowledge is explicitly described by the quintet Novice (N), Advanced Beginner (AB), Competent (C), Proficient (P), and Expert (E). The five parties involved reflect on how well each of the subsequent five fuzzy sets represents the learner’s current level of understanding [52]:

- Novice (N): The student has minimal or textbook knowledge of the educational material, without connecting it to the practice. He/she needs close supervision or guidance and has little or no idea of how to deal with complexity.
- Advanced Beginner (AB): The student has basic knowledge of key aspects of the educational material, while straightforward tasks are likely to be performed to an acceptable standard. He/she is able to achieve some steps using his/her own judgment but needs supervision for the overall task.
- Competent (C): The student has good working and background knowledge of the educational material, and results can be achieved for open tasks, though may lack refinement. He/she is able to achieve most tasks using own judgment and copes with complex situations through deliberate analysis and planning.
- Proficient (P): The student has depth of understanding of the educational material, while results are achieved for open tasks. He/she deals with complex situations holistically and has become confident in decision-making.
- Expert (E): The student has authoritative knowledge of the educational material and deep tacit understanding across areas of the domain, while excellence is achieved with relative ease. He/she is able to move between intuitive and analytical approaches with ease.

**Figure 1:**
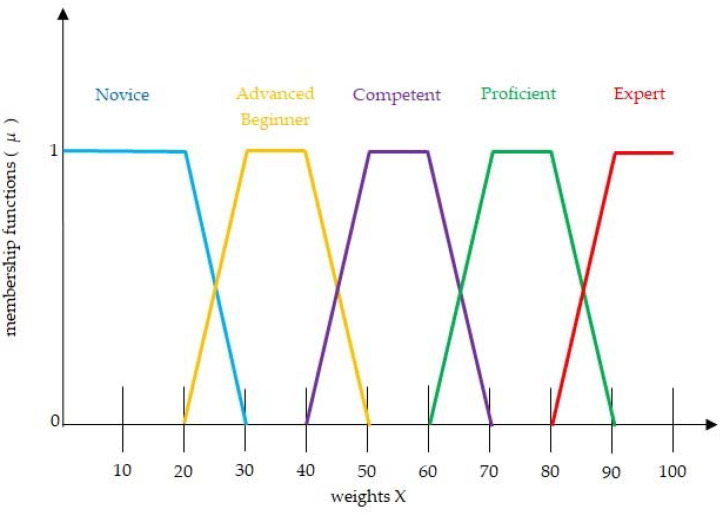
Linguistic scales and fuzzy weights numbers of current knowledge level.

Utilizing the following membership functions, the quintet that describes the student’s present degree of expertise is calculated. The boundary values for each of the aforementioned fuzzy weights, which range from 0 to 1, are expressed using a trapezoidal function (Figure 1). 

**Figure 2:**
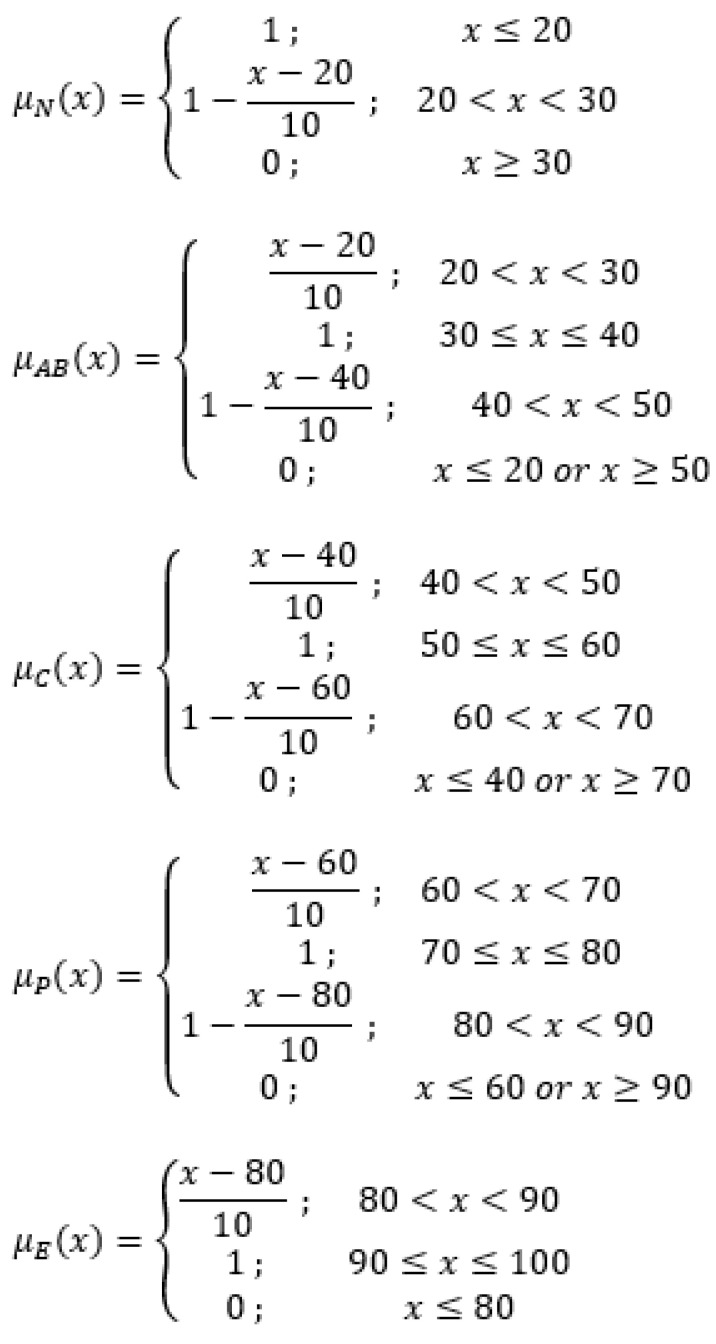
Fuzzy weights membership functions of current knowledge level.

The degree of membership scales from 0 to 1 before flattening out and dropping to 0 at the end. The flattened portion of the trapezoidal function clearly demonstrates that the student’s score (x) belongs in the designated group. The fuzzy weights membership functions are shown in Figure 2.

The student’s level of knowledge is determined by five fuzzy sets (N, AB, C, P, and E), varying from 0 to 1. According to the membership functions presented in Figure 2, a student’s score of 41 equals μ_N_(x) = 0, μ_AB_(x) = 0.90, μ_C_(x) = 0.10, μ_P_(x) = 0, and μ_E_(x) = 0, meaning that the quintet (0, 0.90, 0.10, 0, 0) indicates a student is 90% advanced beginner and 10% competent. In case the student’s score is 48, the resulting values are μ_N_(x) = 0, μ_AB_(x) = 0.20, μ_C_(x) = 0.80, μ_P_(x) = 0, and μ_E_(x) = 0, representing a student who is 20% advanced beginner and 80% competent. The last example of a student’s score of 98 results in μ_N_(x) = 0, μ_AB_(x) = 0, μ_C_(x) = 0, μ_P_(x) = 0, and μ_E_(x) = 1, therefore the quintet (0, 0, 0, 0, 1) indicates a 100% expert student. In any case, the equation μ_N_(x) + μ_AB_(x) + μ_C_(x) + μ_P_(x) + μ_E_(x) = 1 stands.

Eight professors from the Department of Informatics and Computer Engineering utilized the fuzzy weights and the related thresholds at the membership functions. The faculty members were asked to define, in more detail, the technical drawing knowledge levels that students gain during the course throughout the course of an entire semester. All the faculty members have more than 15 years of experience instructing technical drawing in academic contexts, and they can attest to the accuracy of the depiction of students’ knowledge levels.

### 4.2. Decision Making

In this section, the analysis of the rules in combination with the fuzzy weights to adapt the teaching strategy to the students’ knowledge level is presented [28,29,30]. The number of learning activities of each chapter that the student has to learn each time, is dynamically defined according to the current level of knowledge.

The rules design plays an important role for determining the number and the difficulty of the learning activities delivered to the students. The rules have been defined by the aforementioned eight faculty members, who matched each learning activity with the corresponding SOLO level. The set of rules in total is presented in Table 2.

According to these rules, a proficient student who scored 82 per cent is classified as the fourth fuzzy set, as the values of μ_N_(82), μ_AB_(82), μ_C_(82), μ_P_(82), and μ_E_(82) are 0, 0, 0, 0.8, and 0.2, respectively. The delivered learning activities will be:

- No learning activity of SOLO-L1.
- No learning activity of SOLO-L2.
- No learning activity of SOLO-L3.
- Three learning activities of SOLO-L4.
- Two learning activities of SOLO-L5.

## 5. Evaluation Results and Discussion 

### 5.1. Research Population

The current study was evaluated over three courses of an entire academic semester, while students were taking the (a) Computer-Aided-Design, (b) Technical Drawing, and (c) 3D Monument Modelling courses, at an undergraduate curriculum of a public university, located in the nation’s capital. The sample consisted of 148 s, third, and fourth-year undergraduate students, and three educators. All measurements of gender and age were obtained from a randomly chosen sample and had no impact on the outcomes of our study. Table 3 presents the analysis of demographics.

The instructors evenly divided the population into two groups of 74 students. The experimental group (group A) was instructed to run the PARSAT independently, while utilizing the system’s adaptability. For instance, the modeling of students’ knowledge levels enabled them to watch video tutorials of various lengths and rotate 3D objects of various complexity to see and comprehend their structures, and generally engage in various learning activities adjusted to their unique profiles.

The control group (group B) used the same instructional material and exercises, without any customization based on the students’ individual profiles. The students were given instructions on how to carry out the learning activities, which included the same content and approach of the learning activity presented in group A. 

However, the visualization and explanation techniques were different for both groups. The experimental group with AR application could use PARSAT and actually see the system in action on their smartphones and/or tablets and leverage the proposed framework to derive pedagogically meaningful semantics, while group B did not use PARSAT. The educators were involved in the educational procedure in both groups.

### 5.2. Analysis of the Students’ Feedback

At the end of the semester, after the successful completion of all three courses, the two groups were delivered a questionnaire and were asked to respond to the following questions, using a 10-point Likert scale ranging from “Not at all” (0) to “Very much” (10):

- How much did the activities match your level of knowledge? (Q1).
- Was the quantity of the activities used efficient? (Q2).
- Did the activities’ level of complexity enhance your learning? (Q3).

A *t*-test was used to further assess the statistical significance of our findings and respond to the research questions. The statistical importance of the questions 1, 2, and 3 is presented in Table 4, Table 5 and Table 6.

First, we have two conditions, an experimental condition in which students receive the AR-assisted approach and a control condition in which they do not. We compared performance in the two conditions in order to define whether the difference between the two conditions was clear enough or not. In these circumstances, the *t*-test was used to decide whether the difference between the two conditions was real or whether it was due merely to chance fluctuations. For the *t*-test, we were interested in the *p*-value, and we used the *t*-value as an intermediate step to calculate the *p*-value. We state the two conditions, namely null and alternative:

- Condition 0 (null hypothesis): μ1 = μ2 (group A and group B means are equal).
- Condition 1: μ1 ≠ μ2 (group A and group means are not equal).

The 74 participants who used PARSAT (*M* = 8.2, *SD* = 1.1) compared to the 74 participants in the control group (*M* = 6.6, *SD* = 0.4) demonstrated significantly better peak flow scores in the first question, t(239) = 16.9, *p* < *0*.05, as the significance level of the alpha value is a = 0.05 (Table 4). 

The 74 participants of the experimental group (*M* = 8.9, *SD* = 0.8) compared to the participants of group B (*M* = 6.5, *SD* = 0.4) also demonstrated significantly better peak flow scores in the second question, t(270) = 26.8, *p* < *0*.05 (Table 5). 

Finally, the participants of group A (*M* = 9.0, *SD* = 1.0) compared to the participants of group B (*M* = 6.4, *SD* = 0.6) showed much higher peak flow scores in the third question, t(277) = 25.3, *p* < *0*.05 (Table 6). 

This means we have sufficient evidence to say that the means of the two groups are, for all three questions, significantly different, so we reject the null hypothesis.

It is deduced from the analysis of the *t*-test findings (Table 4, Table 5 and Table 6) that the means of the two groups differ statistically significantly regarding the aforementioned questions. More specifically, the provided system outperforms its traditional equivalent in all three terms of the volume of the activities, the complexity of the activities, and the reliability of the activities depending on students’ knowledge levels. These outcomes were anticipated, given that PARSAT uses intelligent techniques to tailor the learning activities to the needs of the students. As a result, a learning environment focused on the needs of the students is provided, and knowledge acquisition and learning outcomes are further enhanced.

### 5.3. Evaluation of the Learning Outcome

In order to compare the learning outcome between the two groups and assess the improvement in their knowledge, we used the pre-test–post-test non-equivalent groups design [53]. In particular, the same pre-test was delivered to each student of group A and group B to evaluate their prior domain knowledge. Both groups of students took the same post-test at the end of the semester, and a paired *t*-test was used to compare the differences and examine whether the students of the experimental group improve more than the students in the control group. Table 7 presents the results of the *t*-test evaluation. 

The analysis of the results of group A (presented in Table 8) from the pre-test (*M* = 4.9, *SD* = 0.9) and post-test (*M* = 6.0, *SD* = 0.9) indicate that the use of the proposed personalized AR application resulted in an improvement in students’ spatial skills, t(147) = −13.8, *p* < 0.05. Furthermore, the Pearson correlation value of r = 0.828 suggests a very strong positive correlation between the pre-test and the post-test scores [54].

The analysis of the results of group B (presented in Table 9) from the pre-test (*M* = 5.0, *SD* = 0.8) and post-test (*M* = 5.5, *SD* = 0.8) indicate that the traditional educational method also resulted in an improvement in students’ spatial skills, t(147) = −6.9, *p* < *0*.05. The correlation between the scores of group B is 0.892, suggesting another very strong correlation.

However, the control group achieved an improvement to a lesser extent than in the experimental group, demonstrating the benefits that our proposed approach brought to students’ learning.

From the analysis of the literature review, it is shown that the current AR applications in engineering education focused only on hardware, not emphasizing intelligent techniques. This study shows a new path toward adaptivity through fuzzification. Furthermore, we create a roadmap to deliver learning activities to students appropriate for their knowledge level.

## 6. Conclusions and Future Work

### 6.1. Conclusions

Our research paper presents a novel instructional strategy by providing students with adaptive learning activities according to the learning theory of the Structure of the Observed Learning Outcomes model. This is achieved by using fuzzy weight-based decision making that defines the students’ knowledge level based on their scores in technical drawing’s domain concepts. As a result, students receive different learning activities based on their level of knowledge.

Regarding the evaluation results, they show high levels of student satisfaction and improvement in their learning outcomes. Specifically, the pre-test and post-test evaluation showed a significant improvement in student outcomes, confirming the pedagogical affordability of the proposed learning method. Lastly, the comparison of the proposed system with the traditional method showed that it outperforms the second one in terms of enhancing the effectiveness of adopted adaptivity and using rule-based training, as well as confirming the efficacy of the teaching method employed in learning activities.

The goal of the current study is to provide students with relevant learning activities while primarily determining their adaptability based on their knowledge level. The introduction of additional fuzzy weights indicating other students’ characteristics, such as categories of mistakes, etc., for increasing the system adaption with the intention to further improve the learning results is a future inquiry resulting from this work.

### 6.2. Theoretical Contributions

This study contributes by exploring fuzzy logic as a tool for assessing students’ knowledge level through AR technology. In our proposed model, we incorporated five fuzzy sets representing the students’ current level of understanding, providing a meaningful addition to the standard training method of engineering students’ spatial skills. 

In doing so, it expands previous studies that have not researched this field in the past. Additionally, our approach tested the students’ group performance and showed that our model is indeed an upgrade in engineering training, helping them to interact better.

### 6.3. Implications in Educational Practice

The results of our study showed that using a mobile AR application personalized to the learner’s knowledge level increased the learning motivation of the students. It is essential for engineers of any field to update their knowledge and train their spatial skills to stay competitive in their field. 

The personalized AR spatial ability training application makes this process more interesting and exciting, and as such, the educational practice becomes more effective. Students are more motivated and more engaged in their training, regardless of their starting level of achievement.

### 6.4. Limitations

The research population involved undergraduate university engineering students. We did not extend the study to non-engineering students and/or learners of a higher age or postgraduate level.

### 6.5. Future Work

The potential for MR, VR, and AR to transform how we interact with the digital environment is high. AR applications seem to be heading toward more practical uses, especially in terms of education. We tested a mobile AR application to train engineering students’ spatial skills. However, in the future, we could explore the impact of VR in delivering spatial skills training. Additionally, a future research field could be the use of MR platforms for practicing the ability to manipulate and interact with an object and improving visualizations skills.

## Figures and Tables

**Table 1:** Learning goals and activities per SOLO level [47,48].

SOLO Level	Learning Goal	Learning Activities	Description of the Activities
Pre-structural (L1)	Students get information on the subject	Define concepts List items Match information Name facts	Introduction to Technical Drawing: A history and current importance of drawing are presented. Students are asked to illustrate the significance of drawing by presenting applications and reports of both good and negative uses of the skill
Unistructural (L2)	Students define, recognize, name, sketch, reproduce, recite, follow simple instructions, calculate, reproduce, arrange, find	5. Identify content to be memorized, show examples 6. Provide disciplinary context 7. Mnemonics in groups 8. Repetition of procedures 9. Games 10. Repetitive testing and matching 11. Peer testing (one student asks, one answers)	Setting up a model space in CAD software by defining limits, grid, snap, layers, and object snap. Video tutorials on standard viwes, views alignment, completion of activity sheet, and setting up the model space. Border creation with a completed title block to be used for all future drawings, and drawing templates with all the settings necessary saved within it
Multi-structural (L3)	Students describe, list, classify, structure, enumerate, conduct, complete, illustrate, solve	12. Glossaries of key terms with definitions, classifications, examples to build disciplinary vocabulary 13. Simple laboratory exercises 14. Define terms, compare to glossary 15. Games modelled on Trivial Pursuit, Family Feud	Orthographic drawing creation. Lines, layers. Isometric object drawing. Video tutorials on linetype, lineweight and isometric drawing creation of objects in the activity.
Relational (L4)	Students relate, analyze, compare, integrate, plan, construct, implement, summarize	16. Case studies, simulations, and complex lab exercises 17. Concept maps 18. Research projects and experiential learning cycles 19. Application of theoretical models 20. Reflective journals 21. Student seminars and debates 22. Syndicate groups (each group is part of whole) 23. Problem-Based Learning and Inquiry Learning	Scaling the border and title block to fit the orthographic drawing. Dimensioning an orthographic drawing, Video tutorials on basic dimesioning rules and parts of dimensions Filling in a title block, including Name, Date, Title, Drawing No., and the correct scale. Snapping and Text commands.
Extended abstract (L5)	Students generalize, hypothesize, theorize, predict, judge, evaluate, assess, predict, reason, criticize	24. Self-directed projects involving research, design, application, argumentation, evaluation 25. Case studies involving extensive analysis, debate, reflection, argumentation, evaluation, forecasting 26. Development of a theory or model 27. Experiential learning cycles 28. Problem Based Learning and Inquiry learning	Printing the drawing on 8.5” × 11” paper (letter size) in landscape orientation. Video tutorial on cutting plane, half and full sections. Printer/plotter settings. Export/plot an object that has been drawn in CAD so it can be exported or printed to a variety of other applications. CAD software to create objects that are more precise and sometimes easier to draw in CAD than in other software.

**Table 2:** Decision rules for adaptive instruction.

Fuzzy Weights	L1	L2	L3	L4	L5	Sum of Las
μ_N_ = 1	7	5	0	0	0	12
μ_N_ < 1	6	5	0	0	0	11
μ_AB_ = 1	4	4	2	0	0	10
μ_AB_ < 1 & μ_C_ < 1	3	3	3	0	0	9
μ_C_ = 1	1	1	3	2	1	8
μ_C_ < 1 & μ_P_ < 1	0	1	2	3	1	7
μ_P_ = 1	0	0	1	4	1	6
μ_P_ < 1 & μ_Ε_ < 1	0	0	0	3	2	5
μ_Ε_ = 1	0	0	0	1	3	4

**Table 3:** Demographics.

Measure	Item	Frequency	Percentage (%)
Sample size		148	100.0
Gender	Male	101	68.2
	Female	47	31.8
Age (over 18)	18–19	57	38.5
	20–21	42	28.4
	22–23	36	24.3
	Over 23	13	8.8
Level of prior knowledge	None	129	87.2
	Technical background	19	12.8
Computer skills	Knowledge of computers at a high level		
Motivation	All students wanted to achieve a high grade at the attended course		

**Table 4:** *t*-test results of Q1.

	Group A	Group B
Mean	8.236	6.581
Variance	1.053	0.368
Observations	74	74
Hypothesized Mean Difference	0	
df	239	
t Stat	16.900	
P (T ≤ t) one-tail	<0.001	
t Critical one-tail	1.651	
P (T ≤ t) two-tail	<0.001	
t Critical two-tail	1.970	

**Table 5:** *t*-test results of Q2.

	Group A	Group B
Mean	8.899	6.500
Variance	0.772	0.415
Observations	74	74
Hypothesized Mean Difference	0	
df	270	
t Stat	26.785	
P (T ≤ t) one-tail	<0.001	
t Critical one-tail	1.651	
P (T ≤ t) two-tail	<0.001	
t Critical two-tail	1.969	

**Table 6:** *t*-test results of Q3.

	Group A	Group B
Mean	9.047	6.378
Variance	1.025	0.618
Observations	74	74
Hypothesized Mean Difference	0	
df	277	
t Stat	25.333	
P (T ≤ t) one-tail	<0.001	
t Critical one-tail	1.650	
P (T ≤ t) two-tail	<0.001	
t Critical two-tail	1.969	

**Table 7:** *t*-test results of pre-test and post-test.

	Group A	Group B
Pre-test Mean	4.973	5.014
Post-test Mean	6.027	5.493
Difference	1.054	0.480
Standard Deviation	0.932	0.837
Pearson Correlation	0.828	0.892
t Stat	−13.765	−6.974
*p*-value	<0.001	<0.001

**Table 8:** *t*-test: Paired two samples for means of group A.

	Pre-test	Post-test
Mean	4.973	6.027
Variance	2.054	2.748
Observations	74	74
Pearson Correlation	0.828	
Hypothesized Mean Difference	0	
df	147	
t Stat	−13.765	
P (T ≤ t) one-tail	<0.001	
t Critical one-tail	1.655	
P (T ≤ t) two-tail	<0.001	
t Critical two-tail	1.976	

**Table 9:** *t*-test: Paired two samples for means of group B.

	Pre-test	Post-test
Mean	5.014	5.493
Variance	2.068	3.272
Observations	74	74
Pearson Correlation	0.892	
Hypothesized Mean Difference	0	
df	147	
t Stat	−6.974	
P (T < = t) one-tail	<0.001	
t Critical one-tail	1.655	
P (T < = t) two-tail	<0.001	
t Critical two-tail	1.976	

## Data Availability

Data are available on request.
